# Relationship between Hormonal Changes and Self-Perceived Halitosis in Females: A Cross-Sectional Study

**DOI:** 10.3390/healthcare11010043

**Published:** 2022-12-23

**Authors:** Hamad Alzoman, Lamees Alssum, Mohammad Helmi, Lama Alsaleh

**Affiliations:** 1 Department of Periodontics and Community Dentistry, College of Dentistry, King Saud University, P.O. Box 60169, Riyadh 11545, Saudi Arabia; 2 College of Dentistry, King Saud University, Riyadh 11545, Saudi Arabia

**Keywords:** halitosis, malodor, menstrual cycle, oral health

## Abstract

Halitosis is a growing concern for patients seeking dental treatment. Women experience hormonal changes throughout different phases of their life. These changes may affect volatile organic compound (VOC) production and can be linked to halitosis. The aim of this study was to evaluate the effect of hormonal changes on self-perceived halitosis in a sample of females using a self-administered questionnaire. This study used a cross-sectional observational design. The questionnaire was distributed electronically through Google forms. A total of 1089 participants completed the questionnaire. Almost 50% of participants were below the age of 25; and 12% were of menopausal age (defined as 45 years and older). Most participants reported having regular menstrual cycles (63.5%) while only 20% reported having hormonal disturbances. Almost 62% of the sample reported that they notice halitosis on themselves with 27.6% indicating their perception of halitosis occurred at different times of the month. Around 12% of the sample thought that a relationship between halitosis and their menstrual cycle existed with 4.6% reporting halitosis during menstruation. The present study found that regularity of menstrual cycle and hormonal disturbances can affect females’ self-perceived halitosis and the prevalence of oral health symptoms.

## 1. Introduction

Halitosis is a growing concern for both patients seeking dental treatment and dental professionals. Halitosis, or what is referred to simply as “bad breath”, has a prevalence ranging from 6% to 50%, in different populations [1,2,3,4]. In Saudi Arabia, the prevalence of 20% to 60 % has been reported [5,6]. Halitosis can have serious effects on an individual’s personal, professional, and social life. 

Halitosis can originate from intraoral or extraoral sources. In the majority of cases, up to 85%, the cause is from the oral cavity [7]. Periodontal diseases, odontogenic infections, dental caries, and tongue coating are some of the common causes of bad breath [8]. Systemic conditions, such as diabetes, gastrointestinal tract (GIT), hepatic, renal, and/or respiratory diseases and conditions, can also cause halitosis [8,9]. 

Volatile organic compounds (VOCs) are produced intraorally and from different parts of the body and can be detected in the exhaled breath [7,10,11]. In a disease state, a shift in the body’s pathophysiology can lead to a change in VOC production that leads to halitosis [12,13]. The most commonly identified VOCs in the exhaled air are volatile sulfur compounds (VSCs), including hydrogen sulfide (H_2_S), methyl mercaptan (CH_3_SH), and dimethyl sulfide (CH_3_)_2_S) [7].

Women experience hormonal changes throughout different phases of their life. These hormonal fluctuations affect both body metabolism and cellular biochemistry [14]. During the menstrual cycle (MC), puberty, pregnancy, and menopause, sex hormones (mainly estrogen and progesterone) show different levels. It has been reported that periodontal tissues might be affected due to fluctuations in sex hormones [15]. The periodontium also shows an exaggerated inflammatory response during puberty and pregnancy due to variation in the sex steroid hormones [16]. These changes may affect the VOCs production and can be linked to halitosis [17,18,19,20].

Recent studies have investigated the effect of hormonal changes through the menstrual cycle on VOCs. In a cohort comparing women with natural menstrual cycle to oral contraceptive users, Sukul et al. [20] reported that VOC concentrations followed female sex hormones regulations throughout the menstrual cycle. They showed that the highest concentrations of VOCs were during the ovulation period in women with natural menstrual cycle. Another study [17] reported a high concentration of VSCs during the menstrual and premenstrual phases of the cycle when compared to the follicular phase and a control group of men. On the other hand, Queiroz et al. [19] reported that the concentration of VSCs in the menstrual stage was higher than in the premenstrual and non-menstrual stages. Similarly, a cross sectional study [18] found that 50% of women had severe halitosis during the menstrual but not during premenstrual or follicular phases. This finding agreed with changes in VSCs levels. 

However, the previous work reported a correlation between halitosis measures and menstrual cycle in females but not halitosis perception in relation to menstrual cycle or other hormonal changes. Therefore, the purpose of this cross-sectional study was to evaluate the effect of menstrual hormonal changes on self-perceived halitosis in Saudi female subjects. The null hypothesis stated that different phases of menstrual cycle do not affect self-perceived halitosis and that hormonal changes (irregularity/disturbances) have no significant effect on halitosis. 

## 2. Materials and Methods

### 2.1. Ethical Approval and Study Population

This study was conducted during the period from May to July 2020. Ethical approval was obtained from the College of Dentistry Research Center (CDRC) and Institutional Review Board (IRB), King Saud University, Riyadh, Saudi Arabia (Protocol # E-19-4252). Participation was voluntary, and the purpose of the study was stated clearly on the cover page of the questionnaire. 

### 2.2. Questionnaire

A questionnaire (See Supplementary Materials) was used to assess self-perception of halitosis among Saudi females in relation to hormonal changes. An Arabic version of a previously validated questionnaire was used [5,6]. Minor modifications were made as required. The questionnaire was revised by an expert in the field to ensure the feasibility and practicality of the questionnaire and the recommended changes were made. A pilot sample of 30 subjects was used to validate the questionnaire, and no changes were recommended before resuming the data collection process. The pilot sample data was included in the final analysis. 

The questionnaire included a cover page with an explanation of the study purpose, instructions, and request for participants’ cooperation. It consisted of five parts. The first part collected information about socio-demographic factors, including age, marital status, and occupation. The second part included questions about medical history and details on menstrual cycle and hormonal therapy. The third part was related to the participant’s knowledge about malodor (halitosis), oral hygiene, and health habits. The fourth part was related to the perception of halitosis and its history and social effects. The last part was concerned about the perception of halitosis during the menstrual cycle. The questionnaire was distributed electronically through Google forms. All females who were at least 18 years old were invited to participate. One of the authors was responsible for distributing a secure link to the questionnaire using different social media platforms to reach the required sample. No personal identifying information was collected in the study. In addition, all the data was collected through a secure link and was handled with great caution during the collection and analysis periods. Only team members have access to the questionnaire data. After gathering all responses, data was transported to team member laptop to save it securely and the questionnaire link was inactivated.

### 2.3. Data Analysis

The data were transferred to a computer for analysis using Statistical Package for Social Sciences program for Windows (IBM SPSS Statistics Version 26, Chicago, IL, USA). Simple descriptive statistics as frequency distributions and percentages were calculated for the study variables. The relation of the variables with self-perceived halitosis was also evaluated using chi-square test of independence at 95% confidence (*p* ≤ 0.05).

## 3. Results

A total of 1089 females participated in the questionnaire. Almost 50% of participants were below the age of 25, and 12% were of menopausal age (defined as 45 years and older). Most participants were single (61.3%) and students (49.1%). Descriptive statistics and participants’ socio-demographic data are provided in Table 1 with reported health conditions in Table 2.

The majority of participants reported having a regular menstrual cycle while only twenty percent reported having hormonal disturbances (Table 2). In all, 61.8% of the whole sample reported that they notice halitosis on themselves with 27.6% indicating their perception of halitosis at different times of the month. Further, 12% of the sample reported that they think there is a relation between halitosis and their menstrual cycle, with 4.6% reporting halitosis during menstruation. 

Table 3 presents the association of several questionnaire domains: (1) halitosis self-perception, (2) the regularity of menstrual cycle, (3) reported hormonal disturbances, (4) halitosis social impact, and (5) menstrual cycle halitosis perception with dichotomous category of age, namely, pre- and menopausal (pre-m and m, respectively). Almost equal proportions of pre-menopausal and menopausal women reported having hormonal disturbances. Most menopausal women (65.9%) reported that they do not suffer from hormonal disturbances. In all, 37.4% of pre-menopausal women reported that they do not know whether they have hormonal disturbances. 13.4% of menopausal women indicated that they feel that others avoid them compared to only 7.1% of pre-menopausal women. While both pre-menopausal and menopausal women indicated that halitosis does not affect their personal lives, a higher percentage of menopausal women reported that halitosis does affect their personal lives compared to pre-menopausal women. All these correlations were statistically significant (*p* < 0.05). 

Table 4 presents the association of several questionnaire domains: (1) halitosis self-perception; (2) halitosis social impact; and (3) menstrual cycle halitosis perception with categories of reported regularity of menstrual cycle, namely, regular, irregular, and do not know. When comparing participants with regular menstrual cycles (63.5%) to those with irregular menstrual cycles (25.5%), the groups differed in terms of noticing halitosis on oneself as well as noticing halitosis on oneself in different times of the month, a history of sinusitis, a history of periodontal diseases, halitosis oral symptoms included bleeding on brushing, teeth mobility, xerostomia, bad taste and the presence of tongue plaque. The groups also differed in reporting social measures (being avoided by others). All of these correlations were statistically significant (*p* < 0.05).

**Figure 1:**
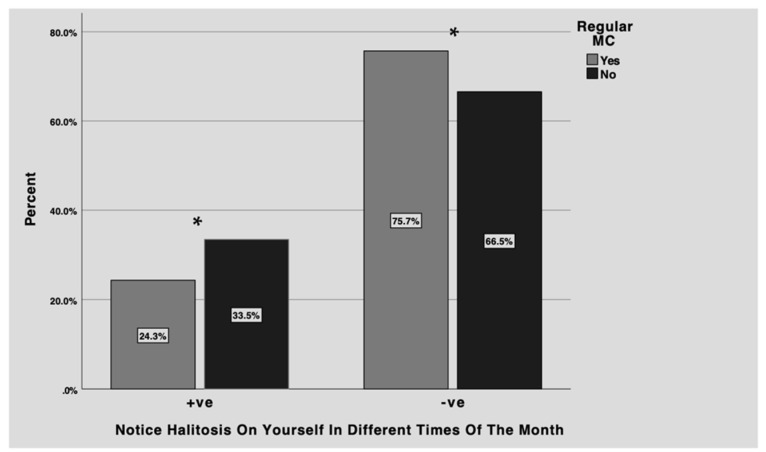
Noticing halitosis on oneself at different times of the month (+ve/yes or −ve/no) of participants who reported regular (grey) versus irregular (black) menstrual cycles. More females with irregular menstrual cycle reported halitosis at different time of the month compared to those with regular menstrual cycles. The majority of the female with regular menstrual cycle reported that they do not notice halitosis at different times of the month. The differences between groups were statistically significant (* *p* < 0.05).

Figure 1 presents females who reported noticing halitosis on themselves at different times of the month in relation to menstrual cycle regularity. Around one-third of females with irregular menstrual cycle reported halitosis at different times of the month compared to 24.3% of those with regular menstrual cycles. Most of the female with regular menstrual cycle reported that they do not notice halitosis in different times of the month. The differences between groups were statistically significant (*p* < 0.05).

Table 5 presents the association of several questionnaire domains: (1) halitosis self-perception; (2) history of sinusitis, periodontal diseases, and smoking; (3) halitosis oral symptoms; (4) halitosis social impact; and (5) menstrual cycle halitosis perception with categories of reported hormonal disturbances, namely, suffering from hormonal disturbances, not suffering from hormonal disturbances, and do not know. Comparing participants who reported hormonal disturbances (20%) to participants with no hormonal disturbances (45.5%), the groups were found to differ in terms of noticing halitosis on oneself as well as noticing halitosis on oneself at different times of the month and noticing halitosis on others at different times of the month, a history of periodontal diseases, smoking, halitosis oral symptoms, including bleeding on brushing, teeth mobility, xerostomia, bad taste, and the presence of tongue plaque. The groups also differed in social impact measures (hesitancy in talking to others, avoiding others, being avoided by others, and perception of halitosis having an effect on one’s personal life). All these correlations were statistically significant (*p* < 0.05).

**Figure 2:**
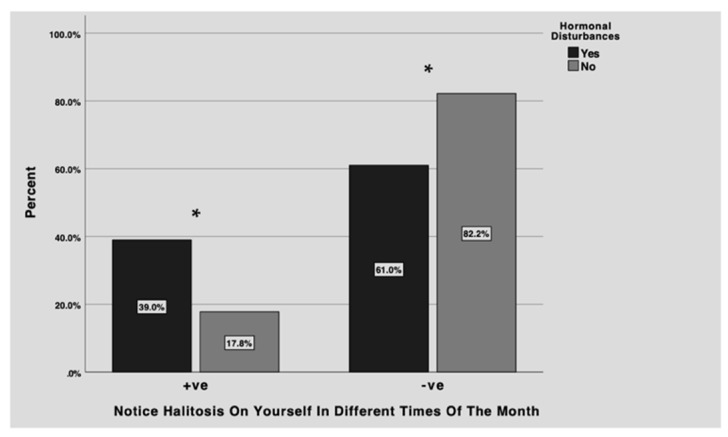
Noticing halitosis on oneself at different times of the month (+ve/yes or −ve/no) of participants who reported having hormonal disturbances (black) compared to participants who reported not having any hormonal disturbances (grey). More females with hormonal disturbances reported halitosis at different times of the month compared to females with no hormonal disturbances. The majority of females who reported no hormonal disturbances, do not notice halitosis on themselves at different times of the month. The results between the two groups were statistically significant (* *p* < 0.05).

Figure 2 presents females who noticed halitosis on themselves at different times of the month in relation to hormonal disturbances. Over one-third of females with hormonal disturbances reported halitosis at different times of the month, while only 17.8% of females with no hormonal disturbances reported halitosis. The majority of females who reported no hormonal disturbances, do not notice halitosis on themselves at different times of the month (82%). The results between the two groups were statistically significant (*p* < 0.05). 

Table 6 presents the association of the social impact and noticing halitosis on yourself at different times of the month. Comparing participants who reported noticing halitosis on themselves at different times of the month to those who do not, the two groups differed in terms of all halitosis social impact measures. All individuals who reported noticing halitosis on themselves at different times of the month tend to hesitate in talking to others, being uncomfortable around others, do not like meeting others, think that they are being avoided by others, and hold the perception that halitosis affects their personal live. The differences between groups were statistically significant (*p* < 0.05).

## 4. Discussion

The aim of this study was to evaluate halitosis perception in relation to menstrual cycle and hormonal changes among Saudi females. Our results shows that around 62% of all females notice halitosis on themselves while 27.5% notice halitosis on themselves at different times of the month. Additionally, the current study found a relationship between menstrual cycle regularity and hormonal disturbances and prevalence of halitosis among females. 33.5% of females with irregular menstrual cycle and 39% with hormonal disturbances reported noticing halitosis on themselves at different times of the month. The present study also found that these hormonal changes have an impact on the females’ social lives. 

In several studies conducted in Saudi Arabia [5,6,21,22,23], the self-perception of halitosis ranged from 16% to 68.5% of participants. Most studies comparing males and females were not conclusive as to which gender has more self-perception of halitosis. No studies comparing only females in terms of premenopausal and menopausal ages, menstrual cycle regularity and irregularity, and hormonal changes have been reported. However, the present study found similar proportions of 61.8% of all females who notice halitosis on themselves.

The age of participants was categorized into pre-menopausal and menopausal as indicators of the likelihood that halitosis will occur based on previous studies reporting that endocrine changes characteristic of the onset of menopause begins around the age of 45 [24]. A comparison of pre-menopausal to menopausal women revealed no statistical significance in terms of halitosis perception.

Women who reported having a regular menstrual cycle reported having lower prevalence of periodontal diseases, bleeding on brushing, and teeth mobility compared to women who reported that their menstrual cycle is not regular. Furthermore, women who reported having hormonal disturbances indicated having a higher prevalence of halitosis oral symptoms compared to women who reported not having any hormonal disturbances. These findings are reflected on the prevalence of self-reported halitosis in general or in different times of the month among females in relation to menstrual cycle and hormonal disturbances. 

Findings in our study are consistent with findings reported in several other studies [17,18,20,25,26]. The effect of the menstrual cycle on halitosis can be attributed to direct and indirect effects. A direct effect is defined as effects on halitosis that are directly related to menstrual cycle and hormonal changes, while indirect effects are defined as effects on halitosis due to modifying variables such as microbiota, saliva, and stress. 

Hormonal changes increase gingival bleeding and the production of proinflammatory cytokines that directly affect halitosis [27,28,29,30]. Furthermore, the direct effects of the menstrual cycle on halitosis can be also explained by the increased production of VSC during menstrual cycle phases [17,18,20,25,26]. In 2008, Calil et al. [17] found that VSC production was higher in menstrual and premenstrual phases when compared with men and the follicular phase. Moreover, in 2010 Kawamoto et al. [25] found that levels of VSC increased by 2.2-fold in the ovulation phase compared to the follicular phase in individuals with periodontitis. 

The indirect effects of the menstrual cycle on halitosis were also reported in the literature [25,26,31,32,33]. Several studies indicated that *Prevotella intermedia*, a microorganism involved in periodontal infections, are at higher levels in ovulation phase than follicular phase [25]. Furthermore, *P. Intermedia* selectively accumulate ovarian hormones estradiol and progesterone that can be used as a substitute for vitamin K, an important nutrient for *P. Intermedia*, resulting in a fostering environment for the microorganism by the increased levels of estrogens and progesterone [32,33]. Moreover, a study conducted by Bostanci et al. [31] in 2021 indicated that an increased abundance of red complex bacteria, an aggregate of bacterial species responsible for severe periodontal diseases, was observed during the follicular phase of the menstrual cycle and was influenced by estradiol levels. The same study reported that hormonal disturbances in females can also affect the normal salivary flow rate resulting in a suppression of the physiological antimicrobial capacity of saliva, which may increase the susceptibility of periodontal diseases in affected women. Another factor that can participate as an indirect effect on halitosis is stress. In 2020, Lima et al. [34] found that academic stress is associated with an increased levels of VSC, alpha-amylase, *Fusobacterium nucleatum*, and total bacteria that can lead to a worsening of oral health outcomes.

After comparing participants who reported noticing halitosis on themselves at different times of the month to those who do not, the two groups differed in terms of all halitosis social impact aspects. Similar to results reported in this current study, de Jongh et al. [35] reported in 2016 that 15–38% of their representative sample always took into account their oral odor when meeting people for the first time. Additionally, the more aware that participants were of their oral odor led them to more likely to maintain a distance when meeting new people, suggesting that self-perception of halitosis affects social interactions. Moreover, a recent systematic review and meta-analysis showed that halitosis is associated with impaired oral health related quality of life [36].

One domain of the questionnaire was aimed at reporting the types and frequencies hormone replacement therapy (HRT) to evaluate its association with halitosis perception. The number of females who indicated taking any hormonal therapy was initially 138. After reviewing each participant’s answer of which HRT they are taking, 42 individuals answered thyroxin, which is not considered an HRT. Therefore, only 96 participants our of 1089 were categorized as using HRT. 

Several variables related to menstrual cycle and hormonal changes were statistically significant. Although the level to which this data is clinically relevant cannot be established, the clinical significance of results maybe most evident in how self-perceived halitosis affects social life for those who reported menstrual cycle- or hormonal-related disturbances.

It is important to elaborate on the sample that reported they do not know respect to certain questionnaire domains. One such example is the response to the question “Do you suffer from hormonal disturbances?” in Table 3. More than 1/3 of the sample (34.5%) indicated that they do know whether they have had hormonal disturbances. This finding might have implications on the statistical significance found for this domain. Although those who reported yes or no to the question were different, the statistical significance might have been influenced by this high percentage of “I don’t know” responses. For example, 13.3% of females who reported that they do not know whether they have hormonal disturbances indicated that they usually notice halitosis on themselves, which was higher than those who reported yes (10.6%) or no (8.3%) with respect to experiencing hormonal disturbances. This finding, however, raises the question: “Are females in need of health education regarding the symptoms of hormonal disturbances?” This finding indicates that there is a high chance that some females are not aware or do not have the ability to perceive if they have hormonal disturbances. Women’s health education regarding the menstrual cycle and hormonal disturbances is necessary to facilitate patient–healthcare provider communication to adequately address the needs of patients. 

Female awareness of good oral hygiene practices in general and taking more considerations during their menstrual cycles may help reduce halitosis during these periods. Additionally, educating female patients through healthcare providers about stress reduction techniques in daily life can help in preventing or reducing oral health symptoms, such as halitosis.

It has been indicated in previous recommendations on the selection of female subjects for breath and mouth odor studies that caution should always be exercised due to cyclical changes in VSC [17,37]. Moreover, measuring the level of stress, salivary flow, and microbiology samples are important variables reported in the literature to be included in clinical trials and should be taken into consideration for their potential indirect effects on halitosis.

Most published research compares halitosis in females with regular menstrual cycles and might exclude females who are undergoing irregular ones. The current study found that females who experience regular menstrual cycles and those who reported no hormonal disturbances have a lower prevalence of oral health symptoms, including halitosis perception, than females with irregular menstrual cycles and hormonal disturbances. Future research is warranted to further investigate factors related to hormonal changes in females and their association with oral health symptoms.

Limitations exist in the current study. This was a cross-sectional study that could be very useful for generating hypotheses. Causality and temporality cannot be assessed due to the inherent nature of the study design. Moreover, self-administered questionnaires have the tendency to introduce recall bias among participants. A more focused group or individual qualitative interview might shed light on personal factors to adequately address halitosis conditions. The sample selected for this study was reached using electronic communication via social media although participation was random among social media users; thus, generalizability to the entire Saudi female population should be approached with caution. Although the findings have implications, further research with repeated measures are necessary to determine magnitude and specific factors related to preventing and treating halitosis. Other factors that could affect self-perceived halitosis (medical condition, oral hygiene measures, the presence of oral and dental disease such as odontogenic infection and dental caries) were not assessed. Furthermore, mouth coverage either for cultural reasons or by using the face mask during the COVID-19 pandemic and its effect on self-perceived halitosis was not evaluated. Although this study has the aforementioned limitations, one strength of this study is its new exploration and focus on females and whether they self-perceive halitosis on themselves during the menstrual cycle and other hormonal changes. 

## 5. Conclusions

The findings of the present study suggested that self-perceived halitosis is related to a female’s menstrual cycle. The regularity of the menstrual cycle and hormonal disturbances can affect females self-perceived halitosis. Future research is warranted to further investigate how factors are related to halitosis. 

## Figures and Tables

**Table 1:** Descriptive statistics and participants’ socio-demographic data.

Demographics	N (%)
**Overall**	1089 (100%)
Age *	
**18–24**	541 (49.7%)
**25–34**	258 (23.7)
**35–44**	158 (14.5)
**45–54**	96 (8.8)
**55 and above**	33 (3%)
Social Status *	
**Single**	668 (61.3%)
**Married**	368 (33.8%)
**Divorced**	44 (4.0%)
**Widow**	6 (0.6%)
Occupation *	
**Student**	535 (49.1%)
**Employed**	352 (32.3%)
**Unemployed/housewife**	199 (18.3%)

* 3 missing.

**Table 2:** Medical and dental history.

**Diabetes**	36 (3.3%)
**Hypertension**	66 (6.1%)
**Sinusitis**	303 (27.8%)
**Respiratory Tract Disease**	84 (7.7%)
**Gastrointestinal Tract Diseases**	197 (18.1%)
**Periodontal Diseases**	130 (11.9%)
**Smoking**	74 (6.8%)
**Regular Menestrual cycle**	691 (63.5%)
**Hormonal Disturbances**	218 (20.0%)
**Polycystic Ovaries Syndrome (PCOS)**	145 (13.3%)
**Hormonal Drugs**	138 (12.7%)

**Table 3:** Association of several questionnaire domains: (1) halitosis self-perception, (2) regularity of menstrual cycle, (3) reported hormonal disturbances, (4) halitosis social impact, and (5) menstrual cycle halitosis perception with dichotomous category of age, namely, pre-menopausal and menopausal (pre = m and m, respectively).

	Age ±			*p*-Value
	N (%)	Pre M	M	
**Overall**	1086 (100%)	957 (88.1%)	129 (11.9%)	
**Notice Halitosis on Yourself**				
**Usually**	114 (10.5%)	107 (11.2%)	7 (5.4%)	0.111
**Sometimes**	557 (51.3%)	484 (50.6%)	73 (56.6%)	
**No**	415 (38.2%)	366 (38.2%)	49 (38.0%)	
**Is Your MC Regular?**				
**Yes**	689 (63.4%)	614 (64.2%)	75 (58.1%)	0.142
**No**	277 (25.5%)	235 (24.6%)	42 (32.6%)	
**I don’t know**	120 (11.0%)	108 (11.3%)	12 (9.3%)	
Do You Suffer from Hormonal Disturbances? *				
**Yes**	215 (19.8%)	189 (19.7%)	26 (20.2%)	0.001
**No**	495 (45.6%)	410 (42.8%)	85 (65.9%)	
**I don’t know**	376 (34.6%)	358 (37.4%)	18 (14.0%)	
**Hesitate Talking to Others (HSI1) §**				
**Yes**	316 (31.6%)	280 (31.7%)	36 (30.5%)	0.432
**No**	455 (45.5%)	403 (45.6%)	52 (44.1%)	
**I don’t know/neutral**	230 (23.0%)	200 (22.7%)	30 (25.4%)	
**Uncomfortable around Others (HSI2)**				
**Yes**	447 (44.7%)	384 (43.6%)	63 (52.9%)	0.072
**No**	411 (41.1%)	366 (41.6%)	45 (37.8%)	
**I don’t know/neutral**	141 (14.1%)	130 (14.8%)	11 (9.2%)	
**Don’t Like Meeting Others (HSI3)**				
**Yes**	226 (22.8%)	205 (23.5%)	21 (17.6%)	0.978
**No**	630 (63.4%)	551 (63.0%)	79 (66.4%)	
**I don’t know**	137 (13.8%)	118 (13.5%)	19 (16.0%)	
Avoided by Others (HSI4) *				
**Yes**	78 (7.8%)	62 (7.1%)	16 (13.4%)	0.001
**No**	640 (64.2%)	582 (66.3%)	58 (48.7%)	
**I don’t know/neutral**	279 (28.0%)	234 (26.7%)	45 (37.8%)	
Halitosis Affect My Personal Life (HSI5) *				
**Yes**	119 (11.9%)	97 (11.0%)	22 (18.8%)	0.011
**No**	763 (76.5%)	686 (78.0%)	77 (65.8%)	
**I don’t know/neutral**	115 (11.5%)	97 (11.0%)	18 (15.4%)	
**Notice Halitosis on Yourself in Different Times of the Month**				
**Yes**	299 (27.5%)	267 (27.9%)	32 (24.8%)	0.529
**No**	787 (72.5%)	690 (72.1%)	97 (75.2%)	
**Notice Halitosis on Others in Different Times of the Month**				
**Yes**	323 (29.7%)	292 (30.5%)	31 (24.0%)	0.151
**No**	763 (70.3%)	665 (69.5%)	98 (76.0%)	

± Distribution of answers within specified age categories; § Responses of HSI1-5 vary for non-respondents; * Statistically significant at alpha = 0.05; MC: menstrual cycle; HIS: halitosis self-perception.

**Table 4:** Association of several questionnaire domains: (1) halitosis self-perception, (2) halitosis social impact, and (3) menstrual cycle halitosis perception with categories of reported regularity of menstrual cycle, namely, regular, irregular, and do not know.

	Is Your MC Regular? ±				*p*-Value
	N (%)	Yes	No	I Don’t Know	
**Overall**	1089 (100%)	691 (63.5%)	278 (25.5%)	120 (11.0%)	
Notice Halitosis on Yourself *					
**Usually**	114 (10.5%)	61 (8.8%)	30 (10.8%)	23 (19.2%)	0.011
**Sometimes**	558 (51.2%)	353 (51.1%)	145 (52.2%)	60 (50.0%)	
**No**	417 (38.3%)	277 (40.1%)	103 (37.1%)	37 (30.8%)	
Sinusitis *					
**Yes**	303 (27.8%)	214 (31.0%)	68 (24.5%)	21 (17.5%)	0.003
**No**	786 (72.2%)	477 (69.0%)	210 (75.5%)	99 (82.5%)	
Periodontal Diseases *					
**Yes**	130 (11.9%)	69 (10.0%)	42 (15.1%)	19 (15.8%)	0.032
**No**	959 (88.1%)	622 (90.0%)	236 (84.9%)	101 (84.2%)	
Bleeding on Brushing *					
**Yes**	431 (39.6%)	249 (36.0%)	120 (43.2%)	62 (51.7%)	0.001
**No**	635 (58.3%)	433 (62.7%)	153 (55.0%)	49 (40.8%)	
**I don’t know**	23 (2.1%)	9 (1.3%)	5 (1.8%)	9 (7.5%)	
Teeth Mobility *					
**Yes**	140 (12.9%)	69 (10.0%)	51 (18.3%)	20 (16.7%)	0.001
**No**	885 (81.3%)	590 (85.4%)	206 (74.1%)	89 (74.2%)	
**I don’t know**	64 (5.9%)	32 (4.6%)	21 (7.6%)	11 (9.2%)	
Xerostomia *					
**Yes**	247 (22.7%)	155 (22.4%)	67 (24.1%)	25 (20.8%)	0.021
**No**	747 (68.6%)	485 (70.2%)	187 (67.3%)	75 (62.5%)	
**I don’t know**	95 (8.7%)	51 (7.4%)	24 (8.6%)	20 (16.7%)	
Bad Taste *					
**Yes**	206 (18.9%)	111 (16.1%)	66 (23.7%)	29 (24.2%)	0.001
**No**	823 (75.6%)	550 (79.6%)	193 (69.4%)	80 (66.7%)	
**I don’t know**	60 (5.5%)	30 (4.3%)	19 (6.8%)	11 (9.2%)	
Plaque on Tongue *					
**Yes**	350 (32.1%)	206 (29.8%)	96 (34.5%)	48 (40.0%)	0.005
**No**	642 (59.0%)	428 (61.9%)	160 (57.6%)	54 (45.0%)	
**I don’t know**	97 (8.9%)	57 (8.2%)	22 (7.9%)	18 (15.0%)	
**Hesitate Talking to Others (HSI1) §**					
**Yes**	318 (31.7%)	190 (30.1%)	88 (34.5%)	40 (34.2%)	0.094
**No**	456 (45.4%)	305 (48.3%)	103 (40.4%)	48 (41.0%)	
**I don’t know/neutral**	230 (22.9%)	137 (21.7%)	64 (25.1%)	29 (24.8%)	
**Uncomfortable around Others (HSI2)**					
**Yes**	450 (44.9%)	274 (43.4%)	119 (46.9%)	57 (48.7%)	0.315
**No**	411 (41.0%)	271 (42.9%)	100 (39.4%)	40 (34.2%)	
**I don’t know/neutral**	141 (14.1%)	86 (13.6%)	35 (13.8%)	20 (17.1%)	
**Don’t Like Meeting Others (HSI3)**					
**Yes**	228 (22.9%)	124 (19.9%)	73 (28.6%)	31 (26.5%)	0.108
**No**	631 (63.4%)	411 (65.9%)	151 (59.2%)	69 (59.0%)	
**I don’t know/neutral**	137 (13.8%)	89 (14.3%)	31 (12.2%)	17 (14.5%)	
Avoided by Others (HSI4) *					
**Yes**	80 (8.0%)	54 (8.6%)	22 (8.7%)	4 (3.4%)	0.004
**No**	640 (64.0%)	422 (67.0%)	90 (35.4%)	76 (65.5%)	
**I don’t know/neutral**	280 (28.0%)	154 (24.4%)	142 (55.9%)	36 (31.0%)	
**Halitosis Affect My Personal Life (HSI5)**					
**Yes**	122 (12.2%)	80 (12.7%)	26 (10.2%)	16 (13.8%)	0.456
**No**	763 (76.3%)	478 (75.9%)	200 (78.7%)	85 (73.3%)	
**I don’t know/neutral**	115 (11.5%)	72 (11.4%)	28 (11.0%)	15 (12.9%)	
Notice Halitosis on Yourself in Different Times of the Month *					
**Yes**	301 (27.6%)	168 (24.3%)	93 (33.5%)	40 (33.3%)	0.005
**No**	788 (72.4%)	523 (75.7%)	185 (66.5%)	80 (66.7%)	
**Notice Halitosis on Others in Different Times of the Month**					
**Yes**	325 (29.8%)	192 (27.8%)	87 (31.3%)	46 (38.3%)	0.055
**No**	764 (70.2%)	499 (72.2%)	191 (68.7%)	74 (61.7%)	

± Distribution of answers within categories of regularity of menstrual cycle; § Responses of HSI1-5 vary for non-respondents; * Statistically significant at alpha = 0.05.

**Table 5:** Association of several questionnaire domains: (1) halitosis self-perception, (2) history of sinusitis, periodontal diseases, and smoking, (3) halitosis oral symptoms, (4) halitosis social impact, (5) menstrual cycle halitosis perception with categories of reported hormonal disturbances, namely, suffering from hormonal disturbances, not suffering from hormonal disturbances, and do not know.

	Do You Suffer from Hormonal Disturbances? ±				*p*-Value
	N (%)	Yes	No	I Don’t Know	
**Overall**	1089 (100%)	218 (20.0%)	495 (45.5%)	376 (34.5%)	
Notice Halitosis on Yourself *					
**Usually**	114 (10.5%)	23 (10.6%)	41 (8.3%)	50 (13.3%)	0.001
**Sometimes**	558 (51.2%)	126 (57.8%)	227 (45.9%)	205 (54.5%)	
**No**	417 (38.3%)	69 (31.7%)	227 (45.9%)	121 (32.2%)	
**Sinusitis**					
**Yes**	303 (27.8%)	68 (31.2%)	138 (27.9%)	97 (25.8%)	0.368
**No**	786 (72.2%)	150 (68.8%)	357 (72.1%)	279 (74.2%)	
Periodontal Diseases *					
**Yes**	130 (11.9%)	40 (18.3%)	34 (6.9%)	56 (14.9%)	0.001
**No**	959 (88.1%)	178 (81.7%)	461 (93.1%)	320 (85.1%)	
Smoking *					
**Yes**	74 (6.8%)	24 (11.0%)	29 (5.9%)	21 (5.6%)	0.022
**No**	1015 (93.2%)	194 (89.0%)	466 (94.1%)	355 (94.4%)	
Bleeding on Brushing *					
**Yes**	431 (39.6%)	99 (45.4%)	163 (32.9%)	169 (44.9%)	0.001
**No**	635 (58.3%)	117 (53.7%)	325 (65.7%)	193 (51.3%)	
**I don’t know**	23 (2.1%)	2 (0.9%)	7 (1.4%)	14 (3.7%)	
Teeth Mobility *					
**Yes**	140 (12.9%)	40 (18.3%)	45 (9.1%)	55 (14.6%)	0.001
**No**	885 (81.3%)	169 (77.5%)	439 (88.7%)	277 (73.7%)	
**I don’t know**	64 (5.9%)	9 (4.1%)	11 (2.2%)	44 (11.7%)	
Xerostomia *					
**Yes**	247 (22.7%)	55 (25.2%)	95 (19.2%)	97 (25.8%)	0.001
**No**	747 (68.6%)	143 (65.6%)	382 (77.2%)	222 (59.0%)	
**I don’t know**	95 (8.7%)	20 (9.2%)	18 (3.6%)	57 (15.2%)	
Bad Taste *					
**Yes**	206 (18.9%)	58 (26.6%)	59 (11.9%)	89 (23.7%)	0.001
**No**	823 (75.6%)	148 (67.9%)	418 (84.4%)	257 (68.4%)	
**I don’t know**	60 (5.5%)	12 (5.5%)	18 (3.6%)	30 (8.0%)	
Plaque on Tongue *					
**Yes**	350 (32.1%)	81 (37.2%)	119 (24.0%)	150 (39.9%)	0.001
**No**	642 (59.0%)	122 (56.0%)	338 (68.3%)	182 (48.4%)	
**I don’t know**	97 (8.9%)	15 (6.9%)	38 (7.7%)	44 (11.7%)	
Hesitate Talking to Others (HSI1) *					
**Yes**	318 (31.7%)	75 (37.1%)	124 (27.9%)	119 (33.3%)	0.033
**No**	456 (45.4%)	88 (43.6%)	222 (49.9%)	146 (40.9%)	
**I don’t know/neutral**	230 (22.9%)	39 (19.3%)	99 (22.2%)	92 (25.8%)	
**Uncomfortable around Others (HSI2)**					
**Yes**	450 (44.9%)	94 (47.0%)	188 (42.2%)	168 (47.2%)	0.387
**No**	411 (41.0%)	86 (43.0%)	197 (44.2%)	128 (36.0%)	
**I don’t know/neutral**	141 (14.1%)	20 (10.0%)	61 (13.7%)	60 (16.9%)	
Don’t Like Meeting Others (HSI3) *					
**Yes**	228 (22.9%)	57 (28.6%)	85 (19.3%)	86 (24.2%)	0.031
**No**	631 (63.4%)	114 (57.3%)	302 (68.5%)	215 (60.4%)	
**I don’t know/neutral**	137 (13.8%)	28 (14.1%)	54 (12.2%)	55 (15.4%)	
Avoided by Others (HSI4) *					
**Yes**	80 (8.0%)	23 (11.4%)	40 (9.0%)	17 (4.8%)	0.001
**No**	640 (64.0%)	112 (55.7%)	308 (69.2%)	220 (62.1%)	
**I don’t know/neutral**	280 (28.0%)	66 (32.8%)	97 (21.8%)	117 (33.1%)	
Halitosis Affect My Personal Life (HSI5) *					
**Yes**	122 (12.2%)	37 (18.5%)	53 (11.9%)	32 (9.0%)	0.011
**No**	763 (76.3%)	139 (69.5%)	348 (78.2%)	276 (77.7%)	
**I don’t know/neutral**	115 (11.5%)	24 (12.0%)	44 (9.9%)	47 (13.2%)	
Notice Halitosis on Yourself in Different Times of the Month *					
**Yes**	301 (27.6%)	85 (39.0%)	88 (17.8%)	128 (34.0%)	0.001
**No**	788 (72.4%)	133 (61.0%)	407 (82.2%)	248 (66.0%)	
Notice Halitosis on Others in Different Times of the Month *					
**Yes**	325 (29.8%)	82 (37.6%)	118 (23.8%)	125 (33.2%)	0.001
**No**	764 (70.2%)	136 (62.4%)	377 (76.2%)	251 (66.8%)	

± Distribution of answers within categories of reported hormonal disturbances; * Statistically significant at alpha = 0.05.

**Table 6:** Association of the social impact and noticing halitosis on yourself in different time of the month.

	Notice Halitosis on Yourself in Different Times of the Month			*p*-Value
	N (%)	Yes	No	
**Oveall**	1004 (100%)	288 (28.7%)	716 (71.3%)	
Hesitate Talking to Others (HSI1) *				
**Yes**	318 (31.7%)	123 (42.7%)	195 (27.2%)	0.001
**No**	456 (45.4%)	94 (32.6%)	159 (50.6%)	
**I don’t know/neutral**	230 (22.9%)	71 (24.7%)	362 (22.2%)	
Uncomfortable around Others (HSI2) *				
**Yes**	450 (44.95)	169 (59.1%)	281 (39.2%)	0.001
**No**	411 (41%)	89 (31.1%)	322 (45%)	
**I don’t know/neutral**	141 (14.05%)	28 (9.8%)	113 (15.8%)	
Don’t Like Meeting Others (HSI3) *				
**Yes**	228 (22.8%)	89 (31.2%)	139 (19.5%)	0.001
**No**	631 (63.4%)	150 (52.6%)	481 (67.7%)	
**I don’t know/neutral**	137 (13.8%)	46 (16.1%)	91 (12.8%)	
Avoided by Others (HSI4) *				
**Yes**	80 (8%)	36 (12.6%)	44 (6.2%)	0.001
**No**	640 (64%)	156 (54.7%)	484 (67.7%)	
**I don’t know/neutral**	280 (28%)	93 (32.6%)	187 (26.2%)	
Halitosis Affect My Personal Life (HSI5) *				
**Yes**	122 (12.2%)	49 (17.2%)	73 (10.2%)	0.005
**No**	763 (76.3%)	200 (70.2%)	563 (78.7%)	
**I don’t know/neutral**	115 (11.5%)	36 (12.6%)	79 (11.1%)	

* Statistically significant at alpha = 0.05.

## Data Availability

Not applicable.

## Supplementary Materials

The following supporting information can be downloaded at: https://www.mdpi.com/article/10.3390/healthcare11010043/s1.
