# Supplementary material for: Relationship between Hormonal Changes and Self-Perceived Halitosis in Females: A Cross-Sectional Study
Source: Healthcare (Basel). 2022 Dec 23;11(1):43. doi: 10.3390/healthcare11010043 (PMC9818922; doi:10.3390/healthcare11010043)
Supplement: Supplementary file 1 [file healthcare-11-00043-s001.zip › healthcare-2004728-supplementary.pdf]

1- Age:
- 18-24
- 25-34
- 35-44
- 45-54
- 55 and above

2- What is your social status:
- Single
- Married
- Divorced
- Widowed

3- What is your occupation:
- Student
- Employed
- Unemployed

4- Do you have diabetes?
- Yes
- No

5- Do you have hypertension?
- Yes
- No

6- Do you have sinusitis?
- Yes
- No

7- Do you have lungs/bronchi diseases?
- Yes
- No

8-Do you have any GIT diseases?
- Yes
- No

9-Do you have any liver diseases?
- Yes
- No

10- Do you have gum diseases?
- Yes
- No

11- Do you smoke?
- Yes
- No

12- If yes, how many cigarettes a day? (Open question)
- Rarely
- Less than 10 per day
- More than 10 per day
- I use vape/electronic cigarettes
- I use Shisha/hookah smoking

13- Do you have a regular menstrual cycle?
- Yes
- No
- I don’t know

14- Do you have any hormonal disorders?
- Yes
- No
- I don’t know

15- Do you have polycystic ovaries syndrome?
- Yes
- No
- I don’t know

16- Are you taking any hormonal treatments including contraceptive pills?
- Yes
- No
- I don’t know

17- If yes, please mention them: (open question)

18- Do you notice bad breaths on other people?
- Yes
- No

19- Do you know the reasons behind bad breaths?
- Yes
- No

20- Gum diseases:
- Strongly agree
- Agree
- I don’t know
- Disagree

- Strongly disagree

21- Dental cavities:
- Strongly agree
- Agree
- I don’t know
- Disagree
- Strongly disagree

22- Coated tongue:
- Strongly agree
- Agree
- I don’t know
- Disagree
- Strongly disagree

23- Dry mouth:
- Strongly agree
- Agree
- I don’t know
- Disagree
- Strongly disagree

24- Smelly food, such as garlic and onions:
- Strongly agree
- Agree
- I don’t know
- Disagree
- Strongly disagree

25- caffeinated beverages, such as coffee and tea:
- Strongly agree
- Agree
- I don’t know
- Disagree
- Strongly disagree

26- Tonsillitis:
- Strongly agree
- Agree
- I don’t know
- Disagree
- Strongly disagree

27- Sinusitis:
- Strongly agree

- Agree
- I don’t know
- Disagree
- Strongly disagree

28- GIT disorders:
- Strongly agree
- Agree
- I don’t know
- Disagree
- Strongly disagree

29- Respiratory disorders:
- Strongly agree
- Agree
- I don’t know
- Disagree
- Strongly disagree

30- others: please mention them: (Open question)

31- There are several treatment options for bad breaths, do you know some of these treatments?
- Yes
- No

32- Toothbrush?
- Yes
- No
- I don’t know

33- Mouthwash?
- Yes
- No
- I don’t know

34- Chewing gum?
- Yes
- No
- I don’t know

35- Tongue scraper?
- Yes
- No
- I don’t know

36- Mouth freshener spray?

- Yes
- No
- I don’t know

37- Visiting the dentist?

- Yes
- No
- I don’t know

38- Other, please mention them: (open question)

39- In your opinion, who is the specialist of choice to diagnose and treat bad breaths?(you can choose more than one answer)

- ENT specialist
- Dentist
- GIT specialist
- Respiratory specialist

40- Have you ever did an examination for bad breaths?

- Yes
- No

41- Have you ever got treated for bad breaths?

- Yes
- No

42- Do you want to be examined for bad breaths by a specialist to know whether you have this problem?

- Yes
- No

43- Do you have bleeding when brushing your teeth?

- Yes
- No
- I don’t know

44- Do you have mobility in your teeth?

- Yes
- No
- I don’t know

45- Do you have dry mouth?

- Yes
- No
- I don’t know

46- Do you have a bad taste in your mouth?

- Yes
- No
- I don't know

47- Do you have coated tongue?

- Yes
- No
- I don't know

48- Do you brush your teeth?

- I don't use
- Rarely
- 1-2 times per week
- 3-4 times per week
- Once a day
- Twice a day

49- Do you use dental floss?

- I don't use
- Rarely
- 1-2 times per week
- 3-4 times per week
- Once a day
- Twice a day

50- Do you use mouthwash?

- I don't use
- Rarely
- 1-2 times per week
- 3-4 times per week
- Once a day
- Twice a day

51- Do you use Miswak?

- I don't use
- Rarely
- 1-2 times per week
- 3-4 times per week
- Once a day
- Twice a day

52- Do you use tongue scraper?

- I don't use
- Rarely

- 1-2 times per week
- 3-4 times per week
- Once a day
- Twice a day

53- Do you use water floss?
- I don’t use
- Rarely
- 1-2 times per week
- 3-4 times per week
- Once a day
- Twice a day

54-Others, please mention them: (open question)

55- Do you notice bad breaths on yourself?
- No
- Sometimes
- Usually

56- If you have bad breaths, when did you notice it?
- I don’t have
- Less than a month
- 1-6 months
- 6-12 months
- More than a year

57- If you have bad breaths, how did you discover it? (you can choose more than one answer):
- I don’t have
- By myself
- By the dentist
- By the general physician
- Someone told me

58- If there is a specific person, please mention who are they? (Open question)

59- Do you notice bad breaths on yourself all the time?
- I don’t notice
- Sometimes
- Always

60- Do you notice bad breaths on yourself when you are hungry?
- I don’t notice
- Sometimes
- Always

61- Do you notice bad breaths on yourself after smoking?

- I don’t notice
- Sometimes
- Always

61- Do you notice bad breaths on yourself after eating smelly food such as garlic and onions?

- I don’t notice
- Sometimes
- Always

62- Do you notice bad breaths on yourself after drinking caffeinated beverages?

- I don’t notice
- Sometimes
- Always

63- Do you notice bad breaths on yourself in the morning?

- I don’t notice
- Sometimes
- Always

64- Other times, such as: (open question)

65- Toothbrush:

- I don’t use
- Never
- Rarely
- Usually
- Always

66- mouthwash:

- I don’t use
- Never
- Rarely
- Usually
- Always

67- Chewing gum:

- I don’t use
- Never
- Rarely
- Usually
- Always

68- Tongue scraper:

- I don’t use
- Never
- Rarely
- Usually
- Always

69- Mouth freshener spray:
- I don’t use
- Never
- Rarely
- Usually
- Always

70- Visiting the dentist:
- I don’t use
- Never
- Rarely
- Usually
- Always

71- others, please mention them: (open question)

72- I hesitate when talking with other people:
- Strongly agree
- Agree
- I don’t know (I’m not sure)
- Disagree
- Strongly disagree

73- I don’t feel comfortable when someone is next to me:
- Strongly agree
- Agree
- I don’t know (I’m not sure)
- Disagree
- Strongly disagree

74- I don’t like meeting other people:
- Strongly agree
- Agree
- I don’t know (I’m not sure)
- Disagree
- Strongly disagree

75- People avoid me because of my bad breaths:
- Strongly agree

- Agree
- I don’t know (I’m not sure)
- Disagree
- Strongly disagree

76- my bad breaths affected my personal life:

- Strongly agree
- Agree
- I don’t know (I’m not sure)
- Disagree
- Strongly disagree

77- other problems? Please mention them (open question)

78- Do you notice on yourself bad breaths during different times of the month?

- Yes
- No

79- Do you notice on other people bad breaths during different times of the month?

- Yes
- No

80- Do you think there is a relationship between halitosis and menstrual cycle?

- Yes
- No

81- If you have bad breaths during your menstrual cycle, when do you notice them? (you can choose more than one answer)

- During the menstrual cycle
- Before the menstrual cycle
- After the menstrual cycle
- I don’t know
